# Cosmic Ray Background Removal With Deep Neural Networks in SBND

**DOI:** 10.3389/frai.2021.649917

**Published:** 2021-08-24

**Authors:** R. Acciarri, C. Adams, C. Andreopoulos, J. Asaadi, M. Babicz, C. Backhouse, W. Badgett, L. Bagby, D. Barker, V. Basque, M. C. Q. Bazetto, M. Betancourt, A. Bhanderi, A. Bhat, C. Bonifazi, D. Brailsford, A. G. Brandt, T. Brooks, M. F. Carneiro, Y. Chen, H. Chen, G. Chisnall, J. I. Crespo-Anadón, E. Cristaldo, C. Cuesta, I. L. de Icaza Astiz, A. De Roeck, G. de Sá Pereira, M. Del Tutto, V. Di Benedetto, A. Ereditato, J. J. Evans, A. C. Ezeribe, R. S. Fitzpatrick, B. T. Fleming, W. Foreman, D. Franco, I. Furic, A. P. Furmanski, S. Gao, D. Garcia-Gamez, H. Frandini, G. Ge, I. Gil-Botella, S. Gollapinni, O. Goodwin, P. Green, W. C. Griffith, R. Guenette, P. Guzowski, T. Ham, J. Henzerling, A. Holin, B. Howard, R. S. Jones, D. Kalra, G. Karagiorgi, L. Kashur, W. Ketchum, M. J. Kim, V. A. Kudryavtsev, J. Larkin, H. Lay, I. Lepetic, B. R. Littlejohn, W. C. Louis, A. A. Machado, M. Malek, D. Mardsen, C. Mariani, F. Marinho, A. Mastbaum, K. Mavrokoridis, N. McConkey, V. Meddage, D. P. Méndez, T. Mettler, K. Mistry, A. Mogan, J. Molina, M. Mooney, L. Mora, C. A. Moura, J. Mousseau, A. Navrer-Agasson, F. J. Nicolas-Arnaldos, J. A. Nowak, O. Palamara, V. Pandey, J. Pater, L. Paulucci, V. L. Pimentel, F. Psihas, G. Putnam, X. Qian, E. Raguzin, H. Ray, M. Reggiani-Guzzo, D. Rivera, M. Roda, M. Ross-Lonergan, G. Scanavini, A. Scarff, D. W. Schmitz, A. Schukraft, E. Segreto, M. Soares Nunes, M. Soderberg, S. Söldner-Rembold, J. Spitz, N. J. C. Spooner, M. Stancari, G. V. Stenico, A. Szelc, W. Tang, J. Tena Vidal, D. Torretta, M. Toups, C. Touramanis, M. Tripathi, S. Tufanli, E. Tyley, G. A. Valdiviesso, E. Worcester, M. Worcester, G. Yarbrough, J. Yu, B. Zamorano, J. Zennamo, A. Zglam

**Affiliations:** ^1^Fermi National Accelerator Laboratory, Batavia, IL, United States; ^2^Argonne National Laboratory, Lemont, IL, United States; ^3^University of Liverpool, Liverpool, United Kingdom; ^4^STFC, Rutherford Appleton Laboratory, Harwell, United Kingdom; ^5^University of Texas at Arlington, Arlington, TX, United States; ^6^CERN, European Organization for Nuclear Research, Geneva, Switzerland; ^7^University College London, London, United Kingdom; ^8^Department of Physics and Astronomy, University of Sheffield, Sheffield, United Kingdom; ^9^University of Manchester, Manchester, United Kingdom; ^10^Universidade Estadual de Campinas, Campinas, Brazil; ^11^Center for Information Technology Renato Archer Campinas, Campinas, Brazil; ^12^Syracuse University, Syracuse, NY, United States; ^13^Universidade Federal do Rio de Janeiro, Rio de Janeiro, Brazil; ^14^Lancaster University, Lancaster, United Kingdom; ^15^Brookhaven National Laboratory, Upton, NY, United States; ^16^Universität Bern, Bern, Switzerland; ^17^University of Sussex, Brighton, United Kingdom; ^18^CIEMAT, Centro de Investigaciones Energéticas, Medioambientales y Tecnológicas, Madrid, Spain; ^19^FIUNA Facultad de Ingeniería, Universidad Nacional de Asunción, San Lorenzo, Paraguay; ^20^University of Michigan, Ann Arbor, MI, United States; ^21^Wright Laboratory, Department of Physics, Yale University, New Haven, CT, United States; ^22^Illinois Institute of Technology, Chicago, IL, United States; ^23^University of Florida, Gainesville, FL, United States; ^24^University of Minnesota, Minneapolis, MN, United States; ^25^Universidad de Granada, Granada, Spain; ^26^Columbia University, New York, NY, United States; ^27^Los Alamos National Laboratory, Los Alamos, NM, United States; ^28^University of Tennessee, Knoxville, TN, United States; ^29^Harvard University, Cambridge, MA, United States; ^30^Colorado State University, Fort Collins, CO, United States; ^31^Rutgers University, Piscataway, NJ, United States; ^32^Center for Neutrino Physics, Virginia Tech, Blacksburg, VA, United States; ^33^Universidade Federal de São Carlos, Araras, Brazil; ^34^Universidade Federal do ABC, Santo André, Brazil; ^35^Enrico Fermi Institute, University of Chicago, Chicago, IL, United States; ^36^University of Pennsylvania, Philadelphia, PA, United States; ^37^Universidade Federal de Alfenas, Poços de Caldas, Brazil

**Keywords:** deep learning, neutrino physics, SBN program, SBND, UNet, liquid Ar detectors

## Abstract

In liquid argon time projection chambers exposed to neutrino beams and running on or near surface levels, cosmic muons, and other cosmic particles are incident on the detectors while a single neutrino-induced event is being recorded. In practice, this means that data from surface liquid argon time projection chambers will be dominated by cosmic particles, both as a source of event triggers and as the majority of the particle count in true neutrino-triggered events. In this work, we demonstrate a novel application of deep learning techniques to remove these background particles by applying deep learning on full detector images from the SBND detector, the near detector in the Fermilab Short-Baseline Neutrino Program. We use this technique to identify, on a pixel-by-pixel level, whether recorded activity originated from cosmic particles or neutrino interactions.

## 1. Introduction

Liquid argon time projection chambers (LArTPCs) are high resolution, calorimetric imaging particle detectors. Due to their excellent calorimetric properties and particle identification capabilities (Acciarri et al., 2017b), combined with their scalability to kiloton masses (Abi et al., 2018a), LArTPCs have been selected for a variety of experiments to detect neutrinos in the MeV to GeV energy range. Several 100–1,000 ton-scale LArTPCs have collected substantial amounts of neutrino data [ICARUS at LNGS (Rubbia et al., 2011) and MicroBooNE at Fermilab (Acciarri et al., 2017a)], or been operated in charged particle test beams [ProtoDUNE-SP (Abi et al., 2017) and ProtoDUNE-DP (Abi et al., 2018c) at CERN]. Others are commissioning (ICARUS at Fermilab, Antonello et al., 2015a) or under construction (SBND at Fermilab, Antonello et al., 2015a). Coming later this decade, the Deep Underground Neutrino Experiment, DUNE (Abi et al., 2018b), will be a 10^4^-ton-scale LArTPC neutrino detector built 1.5 km underground in the Homestake Mine in South Dakota.

LArTPCs operating near the Earth's surface [such as SBND, MicroBooNE, and ICARUS comprising the Short-Baseline Neutrino (SBN) program at Fermilab] are susceptible to backgrounds induced by cosmic interactions, which occur at much higher rates than neutrino interactions. In this paper, we present novel techniques for the tagging of cosmic-induced, neutrino-induced, and background-noise pixels, at a single pixel level, using deep convolutional neural networks applied directly to simulated data from the SBND LArTPC detector.

We first present, in section 2, a description of the liquid argon time projection chamber technology, particularly in the context of the SBND experiment where this study is performed. In section 3, we summarize the origin of the problem we solve with convolutional neural networks, including a description of how LArTPC images are created from the raw data for this study. Section 4 summarizes the related work on this challenge, and section 5 describes the details of the dataset used in this study. Sections 6 and 7 describe the design and training of the convolutional neural network, respectively, and section 8 presents a basic analysis based on the trained network.

## 2. The SBND Liquid Argon Time Projection Chamber

The LArTPC is a high resolution, high granularity, scalable particle detector. Many detailed descriptions of LArTPCs are available (Anderson et al., 2012; Antonello et al., 2015b; Acciarri et al., 2017a) but we will summarize the key features here. In this discussion, we will focus on the near detector of the SBN Program at Fermilab, the Short Baseline Near Detector or SBND, since it is the origin of the dataset used here.

A LArTPC is an instrumented volume of purified liquid argon under an approximately uniform electric field. At one side is the source of the electric field, the cathode. At the other side, the anode, are readout channels to detect charge. In SBND, the readout channels are wire-based.

When charged particles traverse the active argon region, they ionize the argon atoms and leave a trail of argon ions and freed electrons. The freed electrons drift under the influence of the electric field toward the sense wires, where they are detected either via induction or directly collected on the sense wires. Each wire is digitized continuously, and the time of charge arrival indicates how far the charge drifted. A very thorough description of the mechanisms and signal processing for wire-based TPCs can be found in Adams et al. (2018a,b).

**Figure 1:**
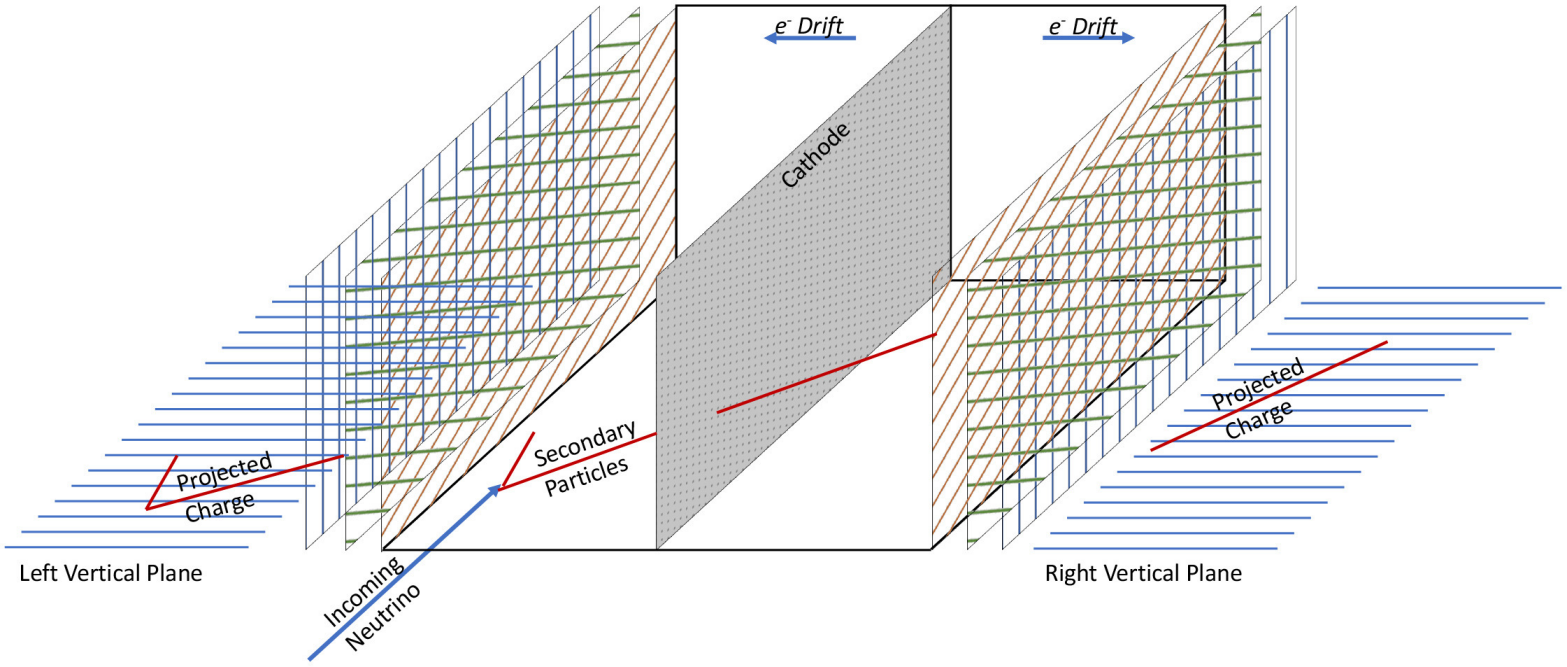
An illustration of the SBND TPC used in this work. In this image, a neutrino interacts in the left TPC, and the outgoing particles cross the central cathode into the right TPC. The top-down projection images (vertical wire planes) are shown, which are combined into one image as seen in Figure 4.

**Figure 2:**
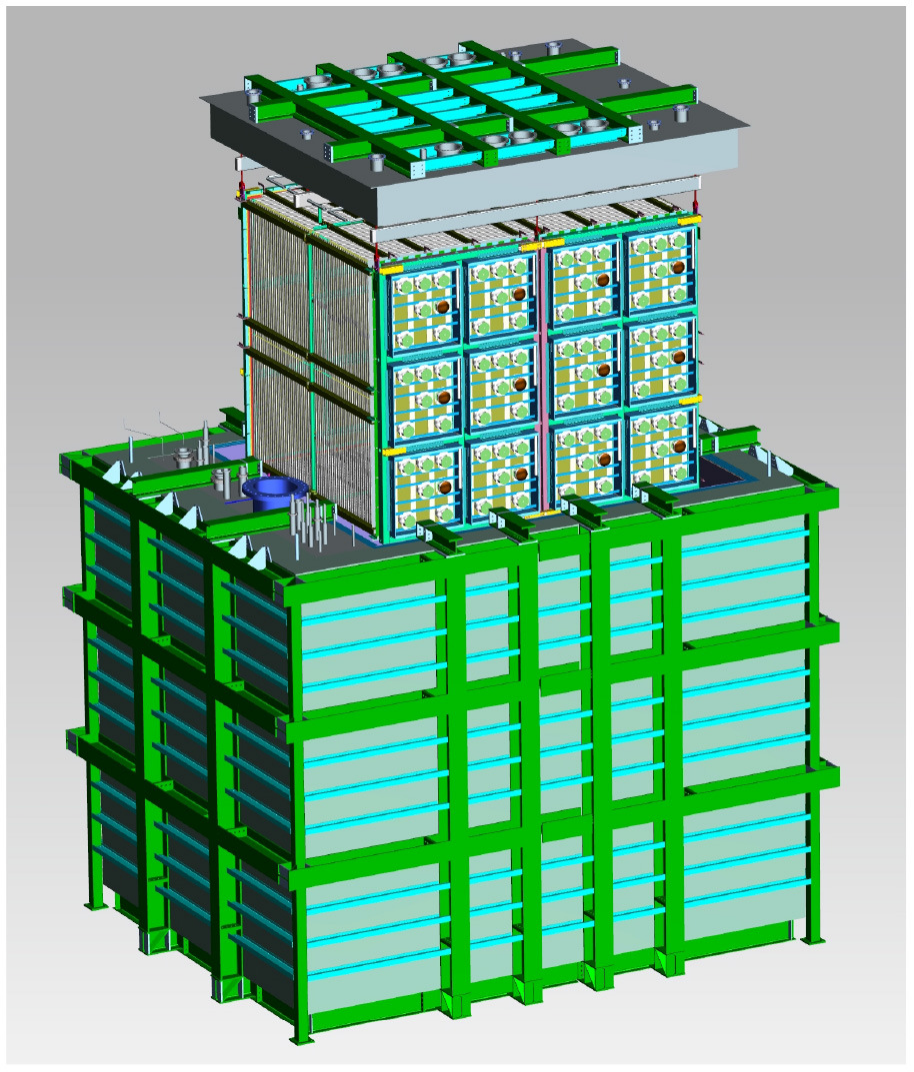
Engineering diagram of the SBND LArTPC and its surrounding subsystems. Here, the TPC is shown lifted above the cryostat for clarity.

The SBND detector is a dual drift TPC, with a central, shared cathode and two anodes, one at each side of the detector (see Figure 1). The vertical wire planes each have 1,664 wires (plane 2 in images in this work), and each of the induction planes (angled at ±60°, planes 0 and 1 in this work) have 1,984 wires (Acciarri et al., 2020). Each TPC is ~5 m long, 4 m high, and 2 m in the drift direction—for a total width of ~4 m. The entire TPC is located within a cryogenic system, as seen in Figure 2.

SBND is also surrounded, nearly entirely, by a solid scintillator-based cosmic-ray muon tracking (CRT) system. The CRT observes the passing of cosmic muons and provides their time of arrival, in principle allowing a veto of some cosmic ray interactions that have no neutrino interactions. Additionally, the interior of the LArTPC detector has a photon detection system to collect the prompt scintillation light that is also generated by charged particles traversing the argon. Both the CRT and photon collection systems could be useful for disentangling cosmic-only and cosmic-with-neutrino events (as described in section 3), but in this work we focus exclusively on analysis of TPC data in the form of 2D images.

SBND is located in the Booster Neutrino Beam at Fermilab, and will observe neutrino interactions in an energy range from a few hundred MeV to several GeV. The SBND detector is under construction at the time of this writing, and results here use simulations based on the design of the detector.

## 3. Problem Description

We seek in this work to remove background activity generated by cosmic particle interactions in the SBND dataset, and in this section we will describe in more detail how the SBND LArTPC operates and why cosmic interactions are problematic.

During typical operation, a LArTPC digitizes the entire detector for a period of time, usually equal to or larger than the time needed for an ionization electron to drift from the cathode to the anode following a “trigger.” A trigger can be caused by any event that would be of interest, such as the arrival of the neutrino beam, the activation of the scintillation detection system above a certain threshold, or a combination of signals from the external CRT system. One digitization of the detector, comprised of the images of each plane for the same time window as well as all auxiliary subsystems, is referred to as an “event.” For a typical LArTPC neutrino detector, the maximum drift time is 1–3 ms.

**Figure 3:**
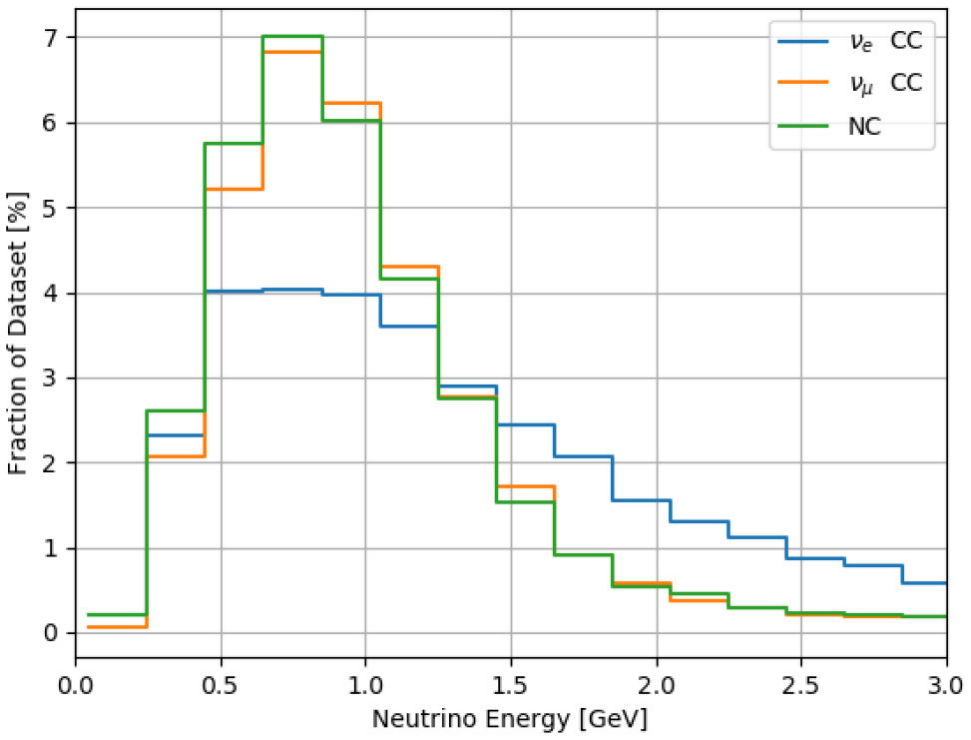
Neutrino energy of interactions produced for this analysis. Most neutral current events are produced by muon-type neutrinos, and so the ν_μ_ CC and Neutral Current energy spectra are similar. The relative populations here are for the dataset used in this paper, while in the neutrino beam the muon neutrino interactions are far more frequent than electron neutrino.

The Booster Neutrino Beam delivers neutrinos to SBND up to 5 times per second, with a neutrino arrival window at the detector that is small (microseconds) compared to the TPC drift time (milliseconds). The histogram in Figure 3 shows the energy of interacting neutrinos simulated in SBND (more details on the simulation are in section 5). The neutrino energies range from tens of MeV to several GeV. When a neutrino interacts with an argon nucleus, it produces an outgoing lepton. For charged current (CC) interactions the outgoing lepton is an electron or muon for an incident electron neutrino or muon neutrino, respectively. For neutral current (NC) interactions the final state lepton is a neutrino, which exits the detector undetected. Both kinds of interactions could also produce other particles such as pions, protons, and neutrons. In liquid argon, at energies relevant to this work (see Figure 3), these particles can travel up to several meters (for energetic muons) or as little as several millimeters (for low energy protons).

**Figure 4:**
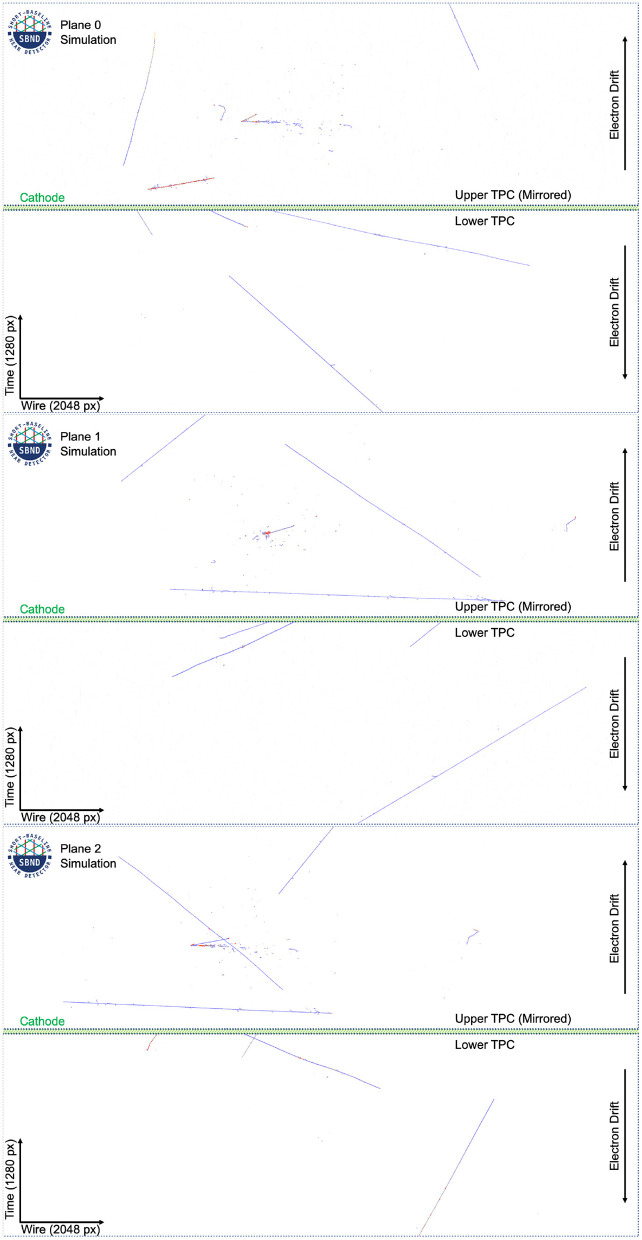
The raw data for one image in the dataset at full resolution. Charge observed is colored with blue for smaller charge depositions and red for larger charge depositions. There is an electron neutrino charged current interaction in the Upper TPC.

During the few millisecond drift time of the ionization electrons, multiple incident cosmic rays will also traverse the TPC. Therefore, a typical event captured in coincidence with the neutrino beam has many cosmic particles visualized in the data, as seen in Figure 4.

As discussed, the scintillation light and CRT auxiliary detectors are useful for rejection of cosmic particles on a whole-image basis, but they do not have granularity to directly remove cosmic-ray induced pixels from TPC data. For example, the photon detectors typically have spatial resolution on the order of tens of cm, while TPC data has a resolution on the scale of millimeters. However, the temporal resolution is significantly better than the TPC. Using this timing information, which can resolve scintillation flashes coincident with the neutrino beam arrival, these detectors can easily reject non-neutrino events that have no scintillation at the right time (the neutrino-beam arrival).

While some cosmic-only events can be rejected with light-only information, for example by requiring a flash of light coincident with the neutrino arrival from the beam, this condition is insufficient to reject every cosmic-only event. In some cases, a neutrino can interact inside of the cryostat but external to the TPC, which is sufficient to cause a detectable flash of light in coincidence with the neutrino arrival. However, no neutrino-induced depositions will be visible in the TPC data, even though all of the standard trigger conditions will have been met.

In another case, since each cosmic interaction also produces scintillation light in the TPC, it is possible for a cosmic particle to produce a flash of light in coincidence with the neutrino beam arrival, even if no neutrino interacts in that event. In this case, the external cosmic ray tagger can identify the cosmic interaction in time with the beam, but these detectors have imperfect coverage and will not distinguish all in-going cosmic muons from outgoing neutrino-produced muons.

Both of these mechanisms cause an event trigger based on a flash of light during the neutrino-arrival window without any neutrino-induced activity in the TPC. And even in events that have a neutrino interaction, the light collection, and cosmic ray tagging subsystems cannot identify the neutrino interaction in the TPC data by themselves. Pattern recognition algorithms applied to TPC data are needed to discern cosmic-induced from neutrino-induced activity. Traditional approaches convert TPC wire data into “hits” (regions of charge above noise threshold) and use geometric relationships to group hits into higher order 2D and 3D multi-hit objects within the TPC images. These objects are treated as particles in the detector and can be further grouped with other associated objects before they are classified as being of cosmic or neutrino origin.

In this work, we take a fundamentally different approach from traditional pattern recognition in LArTPCs by tagging the raw TPC data as cosmic-induced or neutrino-induced on a pixel-by-pixel basis. This tagging, applied early in the analysis of TPC data, can then seed a variety of downstream analysis approaches and provide a significant boost to their performance.

### 3.1. LArTPC Imaging Data

The individual readout “unit” of a LArTPC is the signal along each wire as a function of elapsed time since the trigger or event start. We form 2D images (as seen in Figure 4) from the 1D wire signals as follows. Each column of vertical pixels of the 2D image is two individual wires, one from each TPC, with the 1D signals joined at the cathode in the vertical center of the image. Since the two TPCs drift electrons in opposite directions, away from the central cathode, the 1D signal in the top TPC is inverted compared to the bottom (here, “top” and “bottom” refer to the positions in Figure 4). The signals on each wire are juxtaposed and ordered by increasing wire location, and in this way the collection of 1D readout signals forms a high resolution 2D image.

Each constructed image is effectively a compression of 3D charge locations into a plane that runs perpendicular to every wire in the plane. For the collection plane, with vertically oriented wires, this amounts to a top-down view of the 3D data, where the vertical information is lost in the projection. The other two planes give a different projection, ±60 degrees from vertical, which has the effect of moving the X positions of each charge deposition, while maintaining the Y position, as compared to the vertical projection. Figure 4 shows the 3 wire views from the same 3D interaction in SBND. The images are intrinsically gray scale but have been colored with a color map based on the amount of charge detected per pixel.

The 3D position of a point of charge uniquely determines its location in all three images, and therefore the 3D locations of charge depositions are exactly determined from the 2D images for point-like charge. In practice this inversion task is combinatorically hard with extended objects (and occasionally ambiguous in certain pathological topologies), but some algorithms have made excellent progress (Qian et al., 2018).

## 4. Related Work

The task of pixel level segmentation, or semantic segmentation as it is known, has been explored in depth in computer science journals (Long et al., 2015; Ronneberger et al., 2015), as well as in neutrino physics (Adams et al., 2019). In the UNet architecture (Ronneberger et al., 2015), shortcut connections are introduced to a fully convolutional segmentation network for biological images. The network we present in this paper is similar to the “UNet” architecture in that it has shortcut connections between down-sampled and up-sampled layers of similar resolution. More details of the building blocks and architecture are given in section 6.

In Adams et al. (2019), a modified version of UNet, using residual convolution layers, was deployed to perform pixel-level segmentation of particles based on particle topology; electrons and photons exhibited a broader, “fuzzy” topology when compared to “track”-like particles (protons, muons, pions) which typically are seen as thin, line-like objects^1^. The network was trained on 512 × 512 square images of data from the MicroBooNE detector, and the result was a successful first application of UNet style segmentation techniques to LArTPC neutrino data. Following (Adams et al., 2019), the network described in this paper also applies a series of residual blocks instead of pure convolutions at each image resolution, hence is referred to as “UResNet.”

Additionally, in Dominé and Terao (2020), the authors introduce a spatially sparse, UResNet style architecture for particle-wise segmentation labels in both a 2D and 3D LArTPC-like dataset. Their result is based purely on GEANT4 (Agostinelli et al., 2003) information, meaning that the images did not include the simulation of electronic effects, nor drift-induced effects such as diffusion or absorption of electrons. Nevertheless, this is a novel technique that has broad applicability in neutrino physics. The results presented here use a dense convolutional network, however it is notable that a sparse implementation of the results presented here could deliver gains in performance and computational efficiency.

In MicroBooNE analyses, classical reconstruction techniques are used to reject cosmic ray particles on a particle-by-particle basis, after particles have been “reconstructed” into distinct entities with traditional pattern recognition analyses. For example, in an analysis of charged current muon neutrino (Abratenko et al., 2019) there is still a background of ~35% cosmic or cosmic-contaminated interactions at 50% signal selection efficiency. The results presented here have been developed with the SBND TPC and geometry in mind, but should apply well to the MircoBooNE or ICARUS geometries, also along the Booster Neutrino Beam and part of the SBN Program. In general, the techniques presented here are intended to augment analyses such as Abratenko et al. (2019) to gain better background rejection and better signal efficiency.

## 5. Dataset

The dataset for this application was generated via the larsoft simulation toolkit for LArTPCs (Snider and Petrillo, 2017) utilizing a SBND geometry description and electronics simulation, as of 2018. It was known that the geometry description and electronics simulation for SBND were not finalized at that time, but minor changes to the geometry and electronics response are unlikely to lead to significant changes in the performance we report here. Each event was processed through the simulation of readout electronics and deconvolution, so that the input to the network is identical to data—barring data/simulation issues that are impossible to resolve before operation of the detector.

The drift direction in each plane is digitized at a higher spatial resolution than the wire spacing. For this dataset, the images are downsampled along the drift direction by a factor of 4 to make vertical and horizontal distances have the same scale. To better suit downsampling and upsampling operations, the images are centered horizontally into images with a width of 2,048 pixels, with each pixel representing one wire. The drift direction is 1,260 pixels. Pixels on the right and left, beyond the original image, are set to 0 in both label and input images. The cathode is visible in these images as a green horizontal space in the middle of each image.

Because the images to segment are so large, this work is demonstrating these results on a downsampled version of the images, where each image is at 50% resolution (640 pixels tall, 1,024 pixels wide). The computational challenges leading to this decision are two-fold: first, the GPU memory availability is typically too small to fit the full resolution image during training with all of the intermediate activations required for the gradient calculation. Second, even if the images fit into memory, training on full resolution images takes 4x longer than downsampled images, making model comparisons prohibitively expensive. We anticipate a further publication with full resolution images as the latest computing hardware reduces and eliminates these challenges.

Each interaction in the dataset used here has neutrino interactions simulated with the GENIE software package Andreopoulos et al. (2010) (v2.12.8c), and cosmic backgrounds simulated using CORSIKA (Heck et al., 1998) (v1.7i). The BNB neutrino flux is used to sample neutrinos at the proper energies, however the relative populations of three distinct categories of events (ν_μ_ CC, ν_*e*_ CC, and NC) are balanced in the training set (see Figure 3).

**Figure 5:**
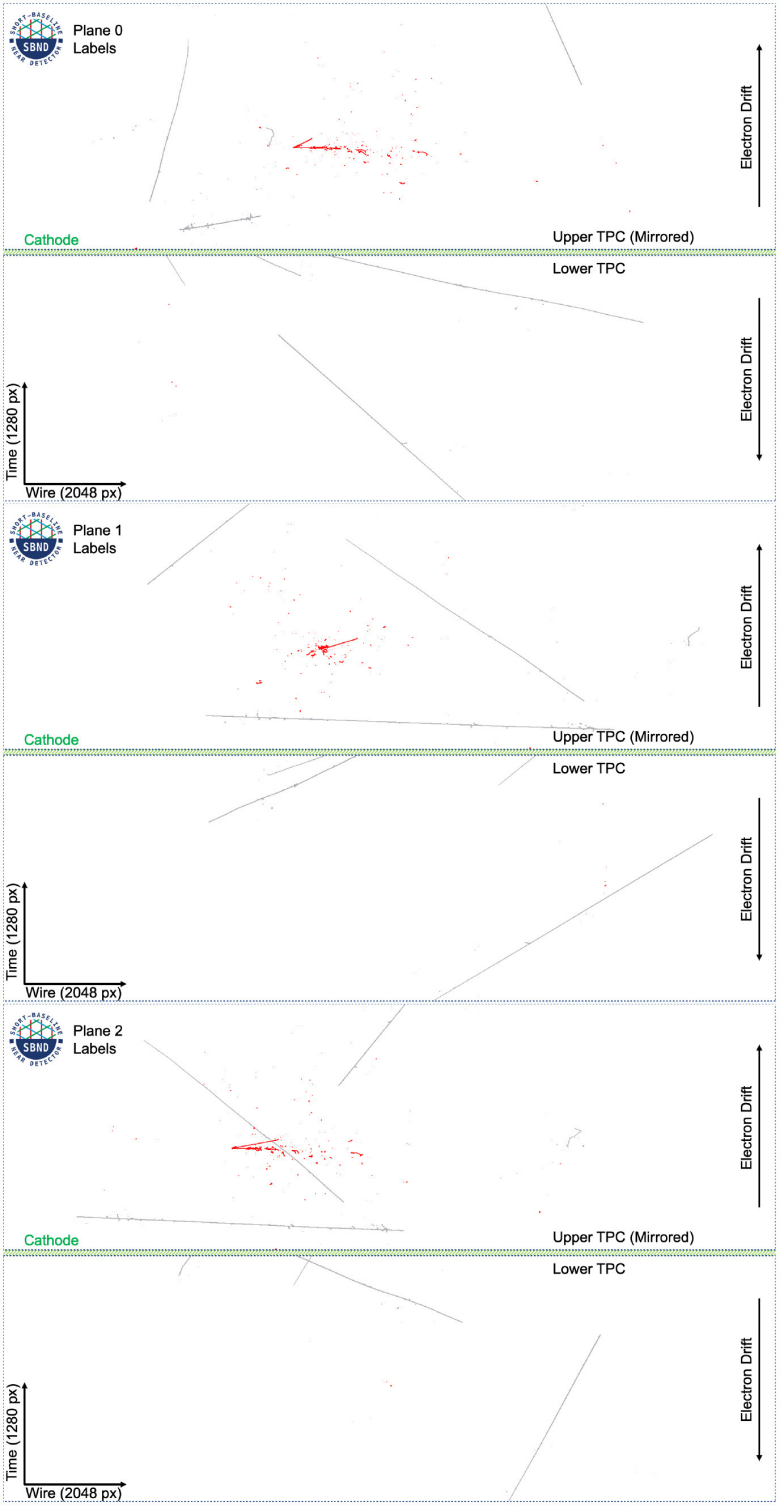
The labels for the images in Figure 4 in the dataset at full resolution. White pixels are background, gray pixels are associated with cosmic particles, and red pixels are associated with a neutrino interaction. Plane 2 shows a case of overlap between cosmic and neutrino pixels.

The label images are created using truth level information from GEANT4 (Agostinelli et al., 2003) (v4.10.3.p01b), where each deposition on a wire is tracked from the particle that created it. Each particle, in turn, is tracked to its parent particle up to the primary particles. All depositions that come from a particle (or its ancestor) that originated with GENIE are labeled as neutrino induced, and all depositions that originated from a CORSIKA particle are labeled as cosmics. In the event of an overlap, as is common, the neutrino label takes precedence. Approximately 50% of all events have an overlap in at least one plane. The label images for the event in Figure 4 can be seen in Figure 5.

## 6. Network Architectures and Implementations

For this work, we present a novel modification of the UResNet architecture for cosmic and neutrino segmentation that aims to meet several criteria:

- Discriminate cosmic pixels from neutrino pixels with high granularity.
- Segment entire events across all planes simultaneously and efficiently.
- Incorporate multi-plane geometrical information.

**Figure 6:**
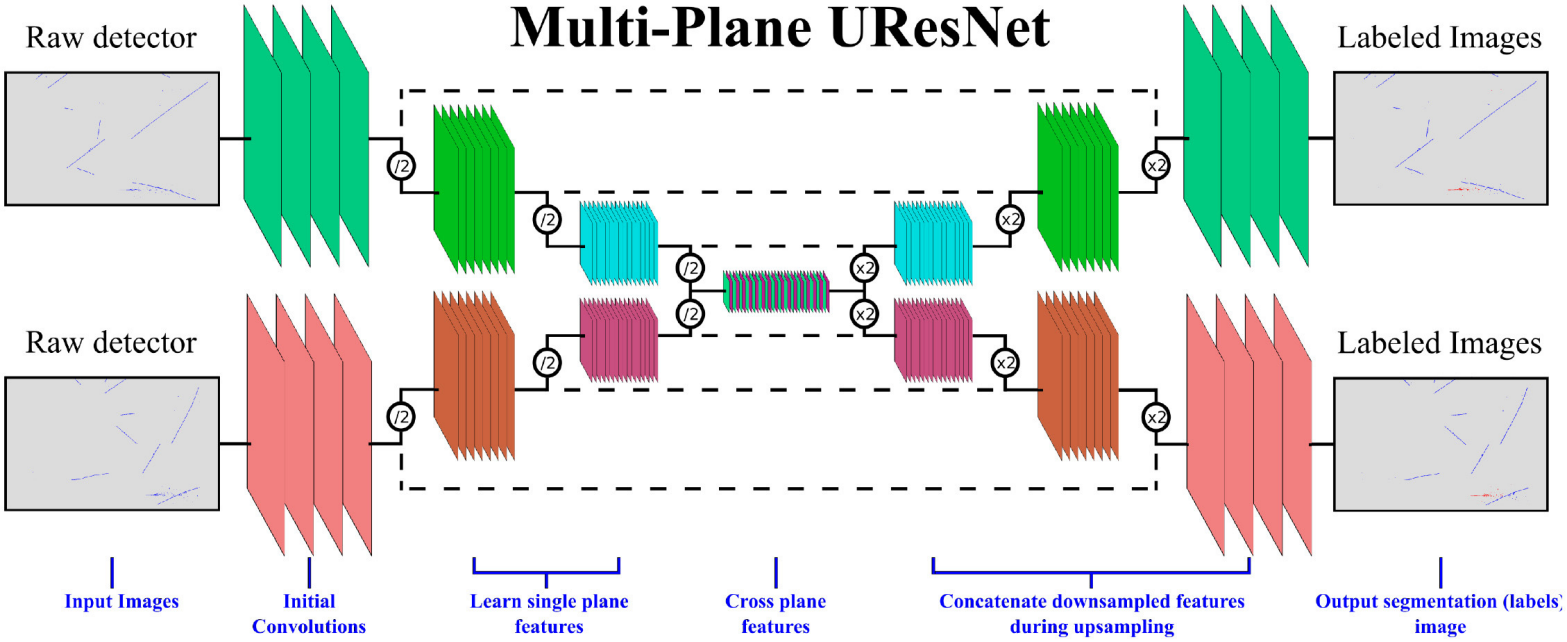
A representation of the multi-plane UResNet architecture. Only two of the three planes are shown in this image for clarity.

To this end, we present a multi-plane, UResNet style architecture as depicted in Figure 6. The input to the network is entire images for each of the three planes, each of which is fed through a segmentation network in the shape of a UResNet. Unique to this work, at the deepest convolutional layer, the per-plane filters are concatenated together into one set of convolutional filters and proceed through convolutions together, in order to learn cross-plane geometrical features. Without this connection at the deepest layer, this network is exactly a “standard” UResNet architecture applied to each plane independently. We see in our experimental results below that without this connection layer, the network does not perform as well. After this, the filters are split and up-sampled independently again.

Because each plane has similar properties at a low level (i.e., particles look similar in each plane, even if the geometric projection is different), convolutional weights are shared across all three planes for up-sampling and down-sampling of the network.

The implementation of the network is available in both TensorFlow (Abadi et al., 2015) and PyTorch (Paszke et al., 2019) on GitHub (Adams, 2021)^2^. The basic building blocks of this network are residual convolutional layers (He et al., 2015). In a residual layer, the input tensor is processed with convolutions, non-linear activations, and (potentially) normalization layers before being summed with the input of residual layer: *R*(*x*) = *x* + *C*(*x*), where *R* is the residual function and *C* represents the convolution layers. In this work, we use Batch Normalization (Ioffe and Szegedy, 2015) as a normalization layer, and LeakyReLU (Maas et al., 2013) as a non-linear activation. Batch Normalization minimizes image to image variance on a whole-image level during training, leading to improved accuracy of deep neural networks. LeakyReLU is a modified version of the rectified linear unit (ReLU), with *f*(*x*) = *x* for *x*>0 and *f*(*x*) = α*x* for *x* < 0, and α small. LeakyReLU preserves gradient information for negative activations (*x* < 0) which proves useful in this network as many pixels are zero, and have negative activation values if any bias terms are negative. While there are many configuration parameters, the baseline model - which we call here Multiplane UResNet—has six levels of depth and the following properties:

- The network operates on each plane independently except at the very deepest layer.
- The first layer of the network is a 7 × 7 convolutional filter that outputs a parameterizable number of filters—the reference models use 16.
- Each subsequent layer in the down-sampling pass takes the previous output and applies two residual blocks, described below, followed by a max pooling to reduce the spatial size. After the max pooling, a bottleneck 1 × 1 convolution increases the number of filters by a factor of 2.
- After the 5th down-sampling pass, the spatial size of the images is (10, 16) with 512 filters in each plane. The images from each plane are concatenated together, and a bottleneck convolution is applied across the concatenated tensor to reduce the number of filters to 256. Then, 5 residual blocks of size 5 × 5 are applied, followed by a 1 × 1 layer to increase the number of filters back to 1,536. The filters are split into three tensors again.
- After the deepest layer, each up-sampling layer takes the output of the corresponding downward pass, adds it to the output of the previous up-sampling layer, and performs two residual blocks with 3x3 convolutions. This pattern of up-sampling/addition/convolutions continues until original resolution is reached.
- Once the original resolution has been restored, a single 1 × 1 convolution is applied to output three filters for each image, where the three filters correspond to the three background classes.

The details of each layer are summarized in Table 1. The residual blocks used in the network mirror those in He et al. (2015), and are the following sequence of operations: convolution, Batch Normalization, LeakyReLU, convolution, Batch Normalization, sum with input, LeakyReLU.

**Table 1:** A description of the multi-plane UResNet architecture used in this work.

**Layer**	**X**	**Y**	**Filters**	**Parameters**	**Operations**
Initial	640	1024	1	416	conv7x7, BN, LeakyReLU
Down 0	640	1024	8	2,576	Res3x3, Res3x3, MaxPool, Bottleneck 8–16
Down 1	320	512	16	10,016	Res3x3, Res3x3, MaxPool, Bottleneck 16–32
Down 2	160	256	32	39,488	Res3x3, Res3x3, MaxPool, Bottleneck 32–64
Down 3	80	128	64	156,800	Res3x3, Res3x3, MaxPool, Bottleneck 64–128
Down 4	40	64	128	624,896	Res3x3, Res3x3, MaxPool, Bottleneck 128–256
Down 5	20	32	256	2,494,976	Res3x3, Res3x3, MaxPool, Bottleneck 256–512
Bottleneck	10	16	1,536	393,984	Concat across planes, bottleneck 1,536–256
Deepest	10	16	256	16,391,680	Res5x5, 5 layers
Bottleneck	10	16	1536	397,824	bottleneck 256 to 1,536, split into 3 planes
Up 5	20	32	256	2,494,208	Interp., Sum w/ Down 5, Bottleneck, Res3x3, Res3x3
Up 4	40	64	128	624,512	Interp., Sum w/ Down 4, Bottleneck, Res3x3, Res3x3
Up 3	80	128	64	156,608	Interp., Sum w/ Down 3, Bottleneck, Res3x3, Res3x3
Up 2	160	256	32	39,392	Interp., Sum w/ Down 2, Bottleneck, Res3x3, Res3x3
Up 1	320	512	16	9,968	Interp., Sum w/ Down 1, Bottleneck, Res3x3, Res3x3
Bottleneck	640	1,024	16	2,552	Bottleneck1x1 to 3 output filters.
Final	640	1,024	3	57	Final Segmentation Maps

To summarize, the network architecture used here is taking state-of-the-art segmentation techniques [“UNet” (Ronneberger et al., 2015) and “UResNet” (Adams et al., 2019)] and enhancing them to learn correlated features across images.

### 6.1. Analysis Metrics

Because of the sparse nature of the images from a LArTPC detector, the per-pixel accuracy does not give good discriminating power to gauge network performance. Simply predicting “background” for all pixels yields a very high accuracy over 99%—even with every “cosmic” and “neutrino” pixel mislabeled. To mitigate this, we calculate several metrics that have proven useful for measuring the performance of a cosmic tagging network:

- **Accuracy** is computed as the total fraction of pixels that are given the correct label by the network, where the predicted label is the highest scoring category in the softmax for that pixel.
- **Non-background Accuracy** is the same as Accuracy above, but computed only for pixels that have a non-zero label in the truth labels. In basic terms, this metric is measuring how often the network is predicting the correct pixel on the parts of the image that matter, as background pixels can easily be identified from their lack of charge.
- **Intersection over Union** (or IoU) is calculated for the neutrino (and cosmic) pixels. This metric uses the set of pixels that are *labeled* (by the simulation) as neutrino (or cosmic) and the set of pixels that are *predicted* (by the network) as neutrino (or cosmic). The metric is the ratio of the number of pixels that are in both sets (intersection) divided by the number of pixels in either set (union). In basic terms, this metric measures how often the network predicts active categories (neutrino, cosmic) on the correct pixels and **only** the correct pixels.

## 7. Training

The network here is trained on a down-sampled version of the full-event images, so each event represents three planes of data at a height of 640 pixels and a width of 1,024 pixels, for a total of 655,360 pixels per plane and 3 planes. Though it would be ideal to train on full-resolution images, this is prohibitive computationally as the network doesn't fit into RAM on current generation hardware.

**Figure 7:**
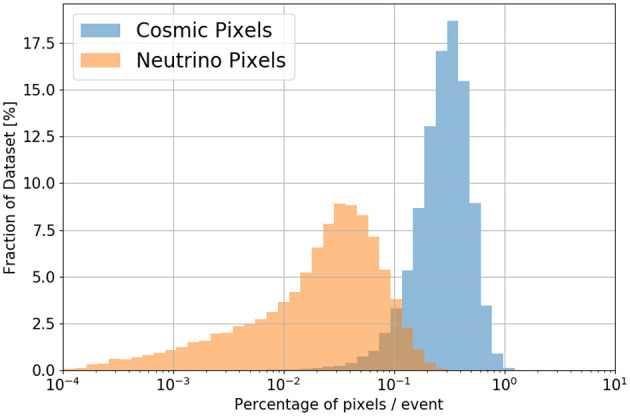
Distribution of pixel occupancies, by label, in this dataset. In general, the cosmic-labeled pixels are <1% of pixels and the neutrino-labeled pixels are <0.3%.

The number of active (non-zero) pixels varies from image to image. In general the number of pixels which have some activity, either from particle interactions or simulated noise, is approximately 11,000 per plane. Of these, approximately 2,300 per plane on average are from cosmic particles, and merely ~250 per plane are from neutrino interactions, on average. See Figure 7 for more details.

To speed up training and ensure the neutrino pixels, which are the most important scientifically, are well-classified, we adopt a weight scaling technique. The loss for each pixel is a three category cross entropy loss, and the traditional loss per plane would be the average over all pixels in that plane. Here, instead, we boost the loss of cosmic pixels by a factor of 1.5, and neutrino pixels by a factor of 10. The final loss is averaged over all pixels in all three planes. We also experimented with a loss-balancing technique where, in each image, the weight for each pixel is calculated so the product of the total weight of all pixels in each category is balanced: *weight*_*background*_ × *N*_*background*_ = *weight*_*cosmic*_ × *N*_*cosmic*_ = *weight*_*neutrino*_ × *N*_*neutrino*_. Experimentally, we find that more aggressive loss boosting of neutrino and cosmic pixels leads to blurred images around the cosmic and neutrino pixels, as those pixels are heavily de-weighted as background pixels. In future studies, we plan to investigate the use of dynamic loss functions such as focal loss (Lin et al., 2017) to allow better balancing of background to significant pixels throughout training.

We report here the performance of several variations of the network, in order to examine the properties of the final accuracy and determine the best network. We test several variations of the network. The baseline model, Multiplane UResNet, is as described above, trained with the mild weight balancing, using an RMSProp (Hinton et al., 2012) optimizer. For variations we train the same network with the following modifications:

- **Concatenated Connections**—instead of additive connections across the “U” (the right-most side of every dashed line), we use concatenation of the intermediate activations (from upsampling and downsampling layers), and 1 × 1 convolutions to merge them.
- **Cross-plane Blocked**—the concat operation blocked at the deepest layer (no cross-plane information), effectively using a single-plane network three times simultaneously.
- **Batch Size × 2**—a minibatch size of 16, instead of 8, is used.
- **Convolutional Upsample**—convolutional up-sampling instead of interpolation up-sampling.
- **Num. Filters/2**—fewer initial filters (8 instead of 16).
- **No Loss Balance**—all pixels are weighted equally without regard to their label.
- **Larger Learning Rate**—the learning rate is set to 0.003 (10x higher).
- **Non Residual**—no residual connections in the down-sampling and up-sampling pass.
- **Adam Optimizer**—unmodified network trained with Adam Optimizer (Kingma and Ba, 2015).
- **Full Balance**—a full loss balancing scheme where each category is weighted such that the sum across pixels of the weights for each category is 1/3.

**Figure 8:**
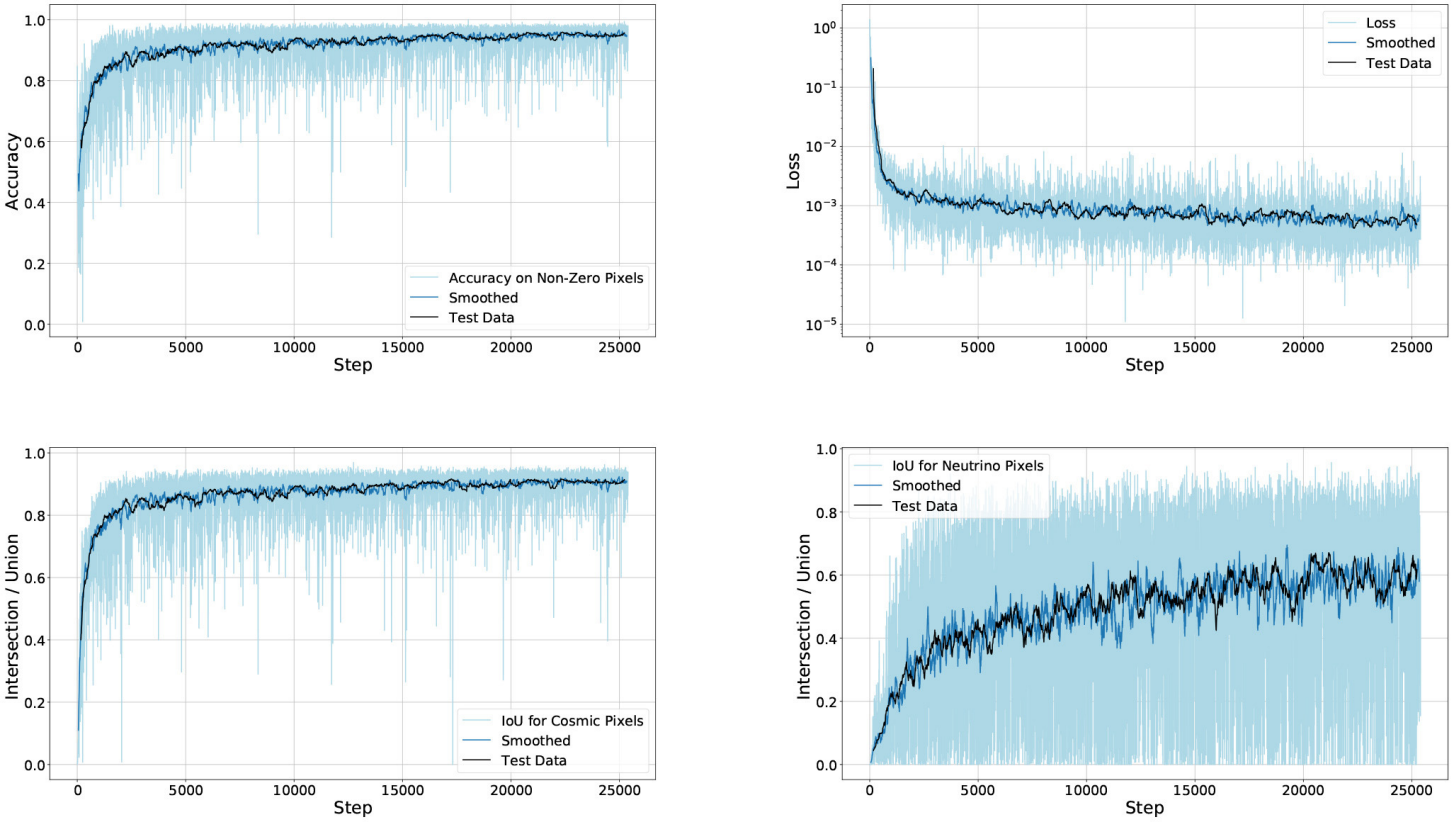
The training progression of the Multiplane UResNet model, trained for 25 k iterations. The light blue curve is the training performance at each step, overlaid with a smoothed representation of the same data, and a smoothed representation of the test set.

All models, except one, are trained with a minibatch size of 8 (× three images, one per plane). The learning rate is set to 0.0003, except for the network that uses a higher learning rate. The other network is trained with a larger batch of 16 images. Due to the memory requirements of this network, a single V100 instance can accommodate only batch size 1. These networks were trained in parallel on 4 V100 devices, using gradient accumulation to emulate larger batch sizes. Figure 8 shows the progression of the metrics while training the Multiplane UResNet model.

In Table 2, we compare the metrics for the different loss schemes and for the network with the concatenate operation blocked. We see good performance in the baseline model, however the models with fully balanced loss and without a concatenate operation are degraded. The full loss balancing exhibits a “blurring” effect around the cosmic and neutrino pixels, since the penalty for over-predicting in the vicinity of those points is minimal. Since nearly half of all events have some overlap between cosmic and neutrino particles, this significantly degrades performance. We also see that using a less extreme loss weighting performs better than no weighting at all, due to the relatively low number of neutrino pixels. Notably, the network with the concatenate connections blocked at the deepest layer (therefore, no cross plane correlation), performs more poorly than the baseline model with every other parameter held constant. Notably, the larger learning rate and use of the adaptive Adam optimizer give poor results with this network.

**Table 2:** A comparison of the performance metrics for the various networks trained.

	**Acc. Non 0**	**Cosmic IoU**	**Neutrino IoU**	**Mean IoU**
Multiplane UResNet	0.951	0.908	0.606	0.757
Concat. connections	0.947	0.898	0.609	0.753
Cross-plane blocked	0.942	0.898	0.571	0.734
Batch size × 2	0.956	0.914	0.698	0.806
Convolution upsample	0.938	0.898	0.539	0.718
Num. filters/2	0.930	0.887	0.457	0.672
No loss balance	0.913	0.882	0.544	0.713
Larger learning rate	0.896	0.852	0.447	0.649
Non residual	0.944	0.904	0.584	0.744
Adam optimizer	0.904	0.852	0.509	0.680
Full balance	0.940	0.720	0.339	0.530

*The best result in each metric is highlighted. The “Mean IoU” is the mean of the cosmic and neutrino IoU values. “Acc. Non 0” refers to the non-background accuracy*.

The larger batch size shows the best performance, including in the average of both IoU metrics. The cosmic IoU is higher than the neutrino IoU due to the difference in difficulty in these labels: many more cosmic pixels implies that errors of a few pixels have a small effect on the cosmic IoU, and a large detrimental effect on the neutrino IoU. We speculate that increasing the batch size further will improve results and will investigate this further with the use of a massive computing system needed to accommodate this large network at a high batch size for training.

As a final comment on the training process, we note that this network is expensive to train and has challenging convergence properties. This has limited the experiments performed on model and training hyperparameters. We expect a future result to investigate hyperparameters in a systematic way. In the following section, we use the model trained with a minibatch size of 16, “Batch Size x2,” as it had the best performance on the test set. Example images of the output of the network are found in Appendix A.

## 8. Analysis Results

**Figure 9:**
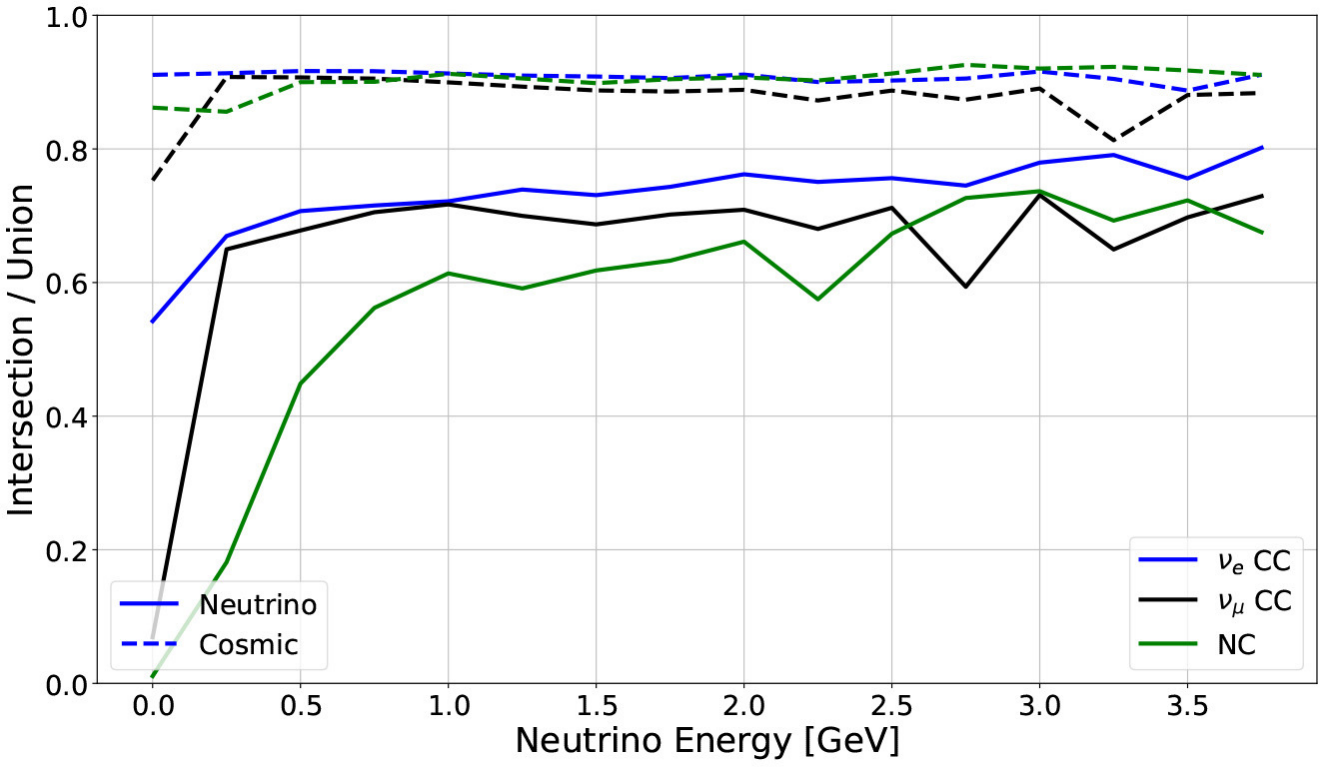
Metric performance across neutrino interaction types, as a function of neutrino energy. The solid lines are the Intersection over Union for the neutrino predicted/labeled pixels, while the dashed lines are the Intersection over Union for the cosmic predicted/labeled pixels. Each color in this plot represents the IoU for all events containing that particular neutrino interaction.

Figure 9 shows the metric performance as a function of neutrino energy for the best performing network, broken out across three kinds of neutrino interactions: electron neutrino charged current, muon neutrino charged current, and neutral current.

To demonstrate the utility of this deep neural network in a physics analysis, we perform a very elementary selection of events. We perform inference on a selection of events from all types of simulated interactions, including events where there is no neutrino interaction.

There are two main objectives of this analysis. First, on an event by event basis, decide if there is a neutrino interaction present in the measured charge using TPC information only. It is expected that any additional information from the light collection or cosmic ray tagging systems will further enhance these results. Second, within an interaction that has been selected as a neutrino interaction, measure the accuracy with which the interaction has been selected from the cosmic backgrounds.

To demonstrate the performance in event-level identification, we apply a simple set of metrics. We require a minimum number of pixels, per image, to be classified as neutrino by the network. Additionally, since the drift direction (Y-axis) of all three images is shared in each event, we apply a matching criterion. Specifically, we compute the mean Y location of all neutrino-tagged pixels in each plane, and we require that the difference in this mean location is small across all three planes.

Quantitatively, we find good results by requiring at least 100 neutrino-tagged pixels per plane, and a maximum separation of mean Y location of 50 pixels across all three combinations of images. With these basic cuts, we observe the selection efficiencies of Table 3. We note that neither 100 pixels per plane, nor a separation distance of 50 pixels, is a well-tuned cut. For some analyses targeting low energy events in the Booster Neutrino Beam, these cuts would be too aggressive. Instead, the desired goal is to demonstrate that the predictive power of this network can be leveraged in a basic event filtering workflow.

**Table 3:** Selection efficiencies for sample cuts using the inference output of the best network.

**Category**	**Efficiency (%)**
ν_*e*_ CC	91.5
ν_μ_ CC	78.6
NC	37.3
Cosmics	91.1 cosmic-only event rejection

The selection efficiencies with these cuts, though not aggressively tuned, do have variation from one type of neutrino interaction to another. The muon-neutrino events are distinguished by the presence of a long muon from the neutrino interaction, while electron neutrino events have no muons and instead an electro-magnetic shower. Since the cosmic particles are primarily, though not entirely, composed of high energy muons, it is not surprising that electron neutrino events are more easily distinguished from cosmic-only events, as compared to muon neutrino events. Additionally, the neutral current events have an outgoing neutrino that carries away some fraction of the energy of the event; on average, these events have much less energy in the TPC and therefore fewer active pixels to use for selection and discrimination of events. Consequently, neutral current events are harder to reject compared to charged current events.

As a comparison to classical techniques, we first note that the metrics presented and used in this paper, which are the right discriminating tools for this machine learning problem, are not studied in the classical analysis. Therefore, a direct comparison to classical results does not exist. We note that in Acciarri et al. (2018), the traditional reconstruction applies a cosmic-muon tagging algorithm in the MicroBooNE detector. This algorithm groups pixels into “clusters” first, and then tags clusters—as a whole—as either cosmic-ray induced or not cosmic-ray induced. The algorithm in Acciarri et al. (2018) quotes a cosmic muon rejection rate of 74%, on average.

While the detectors are different geometries, to first order MicroBooNE and SBND have the same order-of-magnitude flux of cosmic-ray muons. Further, since the cosmic muons account for ~90% of the non-zero pixels, we may speculate that ~26% of cosmic pixels are mislabeled, or ~23% of all non-zero pixels—with the assumption that all neutrino pixels are correctly labeled. In short, though the same metrics are not directly applied in Acciarri et al. (2018) and the detector geometries are slightly different, a rough comparison may be made in the non-zero accuracy metric of 95.6% (this work) to 77% (traditional reconstruction)—in other words, a reduction of 23–4.4% mis-classified pixels on average.

We do not speculate here on final purity for an analysis of this kind on the BNB spectrum of neutrinos at SBND. The final analysis will use both scintillation light and cosmic ray tagger information in addition to the TPC data. However, it is notable that a simple analysis can reduce the cosmic-only interactions by a factor of 10x, and the remaining events have the correct pixels labeled at a 95% non-background accuracy level. We believe this is a promising technique for the SBN experiments.

## 9. Conclusions

In this paper, we have demonstrated a novel technique for pixel level segmentation to remove cosmic backgrounds from LArTPC images. We have shown how different deep neural networks can be designed and trained for this task, and presented metrics that can be used to select the best versions. The technique developed is applicable to other LArTPC detectors running at surface level, such as MicroBooNE, ICARUS, and ProtoDUNE. We anticipate future publications studying the hyperparameters of these networks, and an updated dataset with a more realistic detector simulation prior to the application of this technique to real neutrino data.

## Data Availability Statement

The raw data supporting the conclusions of this article will be made available by the authors, without undue reservation.

## Author Contributions

CAd is the main author and originated the analysis. CAn and DBr served as internal editors. All authors contributed to the article and approved the submitted version.

## Conflict of Interest

The authors declare that the research was conducted in the absence of any commercial or financial relationships that could be construed as a potential conflict of interest.

## Publisher's Note

All claims expressed in this article are solely those of the authors and do not necessarily represent those of their affiliated organizations, or those of the publisher, the editors and the reviewers. Any product that may be evaluated in this article, or claim that may be made by its manufacturer, is not guaranteed or endorsed by the publisher.

## Footnotes

^1^The “fuzzy”ness of electromagnetic particles is due to the electromagnetic cascade or shower of particles initiated by an electron or photon with enough energy to produce more particles.

^2^https://github.com/coreyjadams/CosmicTagger.
