# Supplementary material for: Cosmic Ray Background Removal With Deep Neural Networks in SBND
Source: Front Artif Intell. 2021 Aug 24;4:649917. doi: 10.3389/frai.2021.649917 (PMC8421797; doi:10.3389/frai.2021.649917)
Supplement: Supplementary file 1 [file Data_Sheet_1.pdf]

A Example Images

In this section we present several example images of the network applied to simulated events. We show for each event all three planes the raw, simulated wires, the labels, and the simulated signal where only pixels tagged as neutrino (probability $> 50\%$ are kept.) In each image, we note the mislabeled pixels and subtle failures of the network that degrade the overall accuracy.

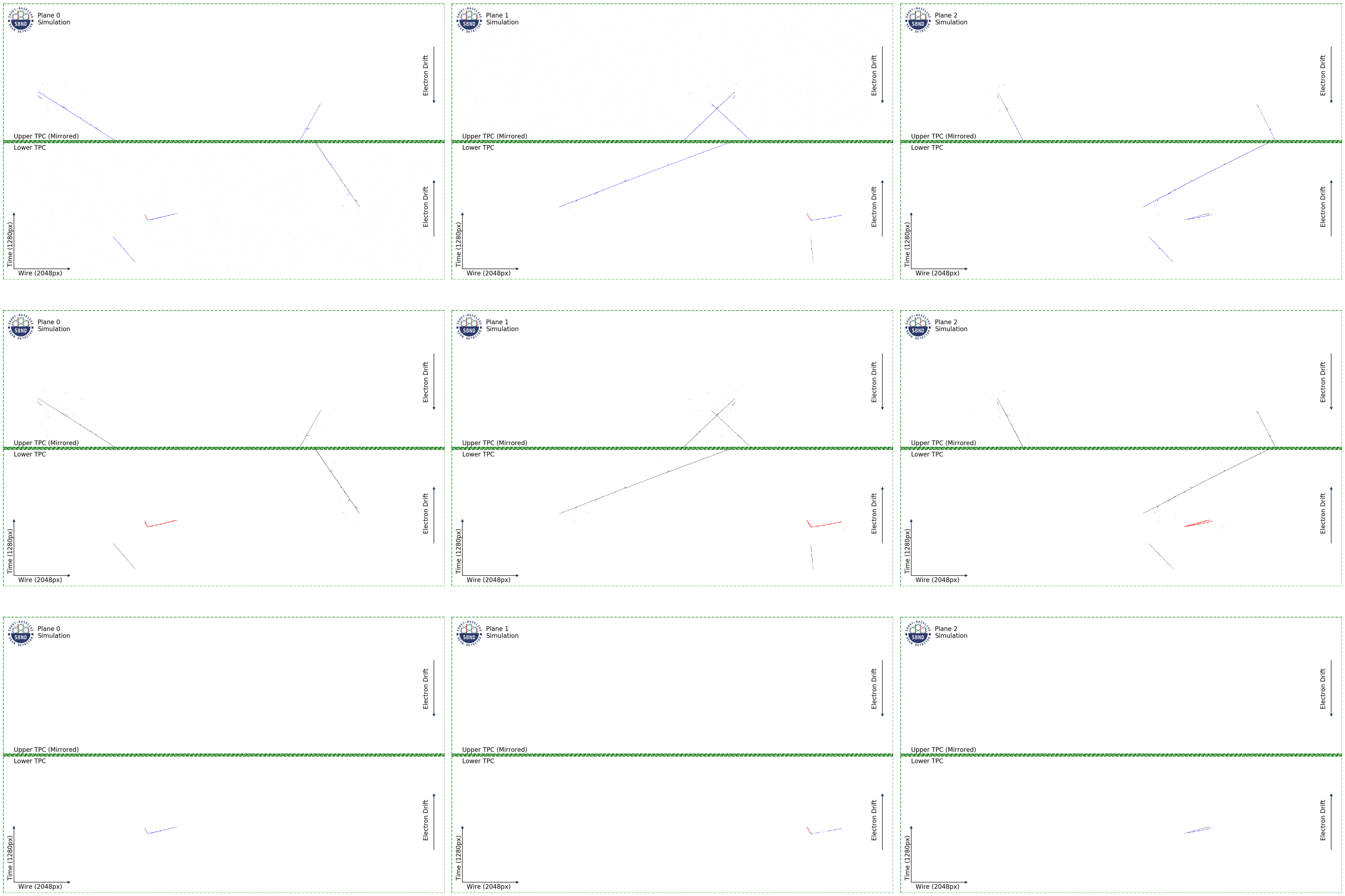

Figure S1: From left to right are the first, second and third projections. From top to bottom are the input images, the label images, and the selected neutrino pixels. The neutrino interaction is well selected except in the middle column, where several pixels are missing.

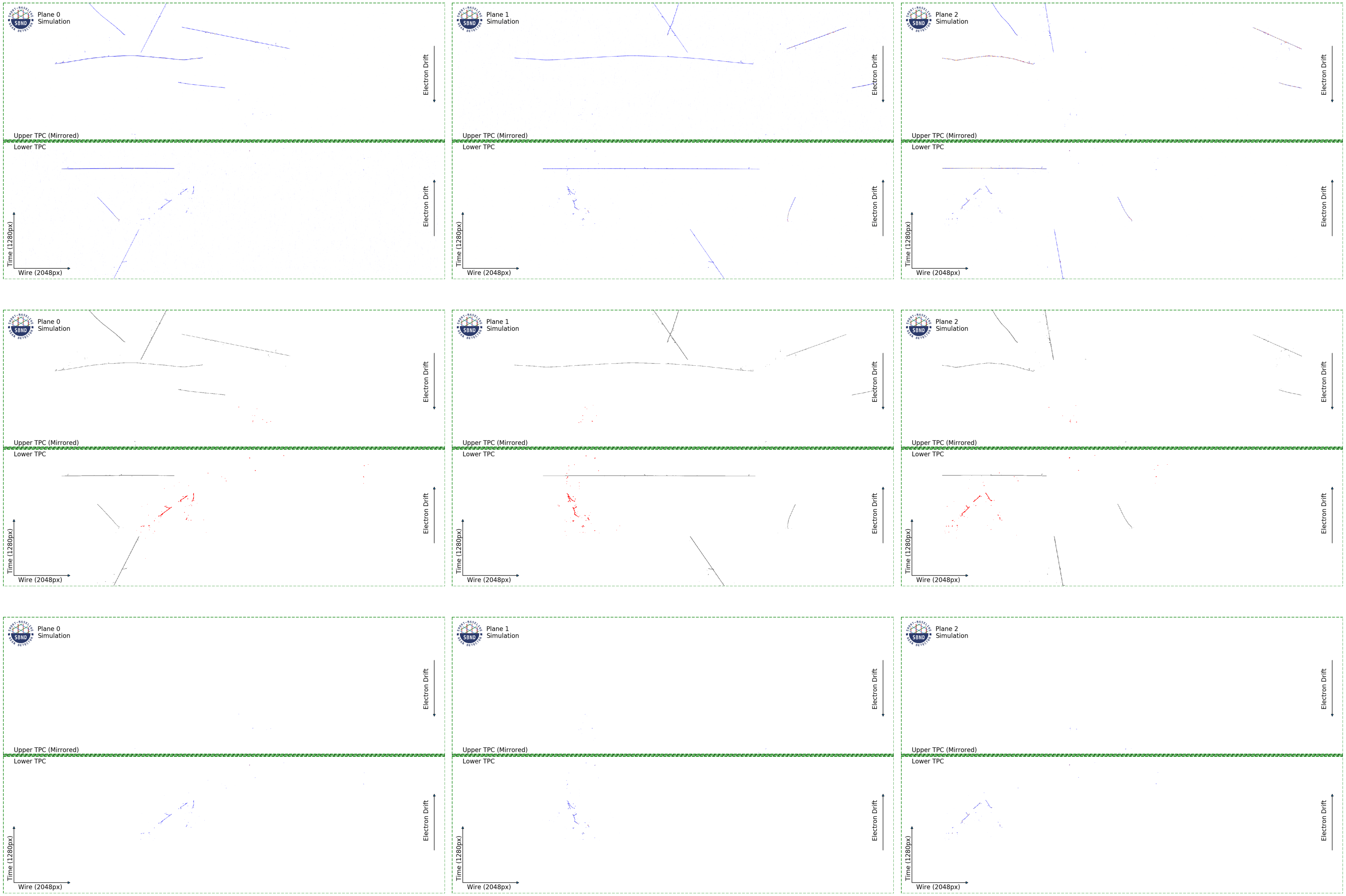

Figure S2: From left to right are the first, second and third projections. From top to bottom are the input images, the label images, and the selected neutrino pixels. The neutrino interaction is almost entirely correct, excepting some of the smaller depositions in the upper TPC.

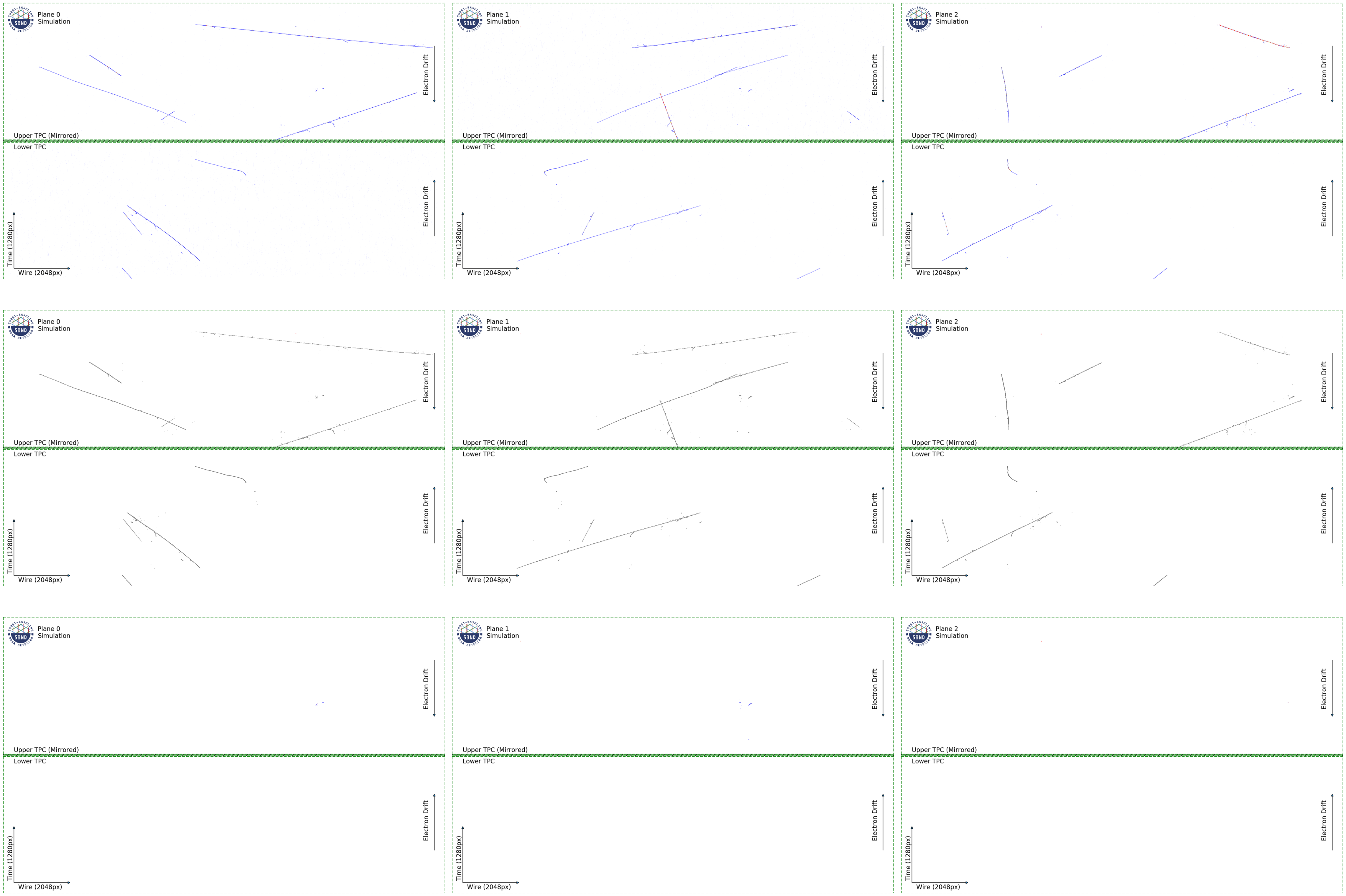

Figure S3: From left to right are the first, second and third projections. From top to bottom are the input images, the label images, and the selected neutrino pixels. This interaction represents a false positive selection, in that a small interaction is selected completely in 2 planes, and partially in a third.

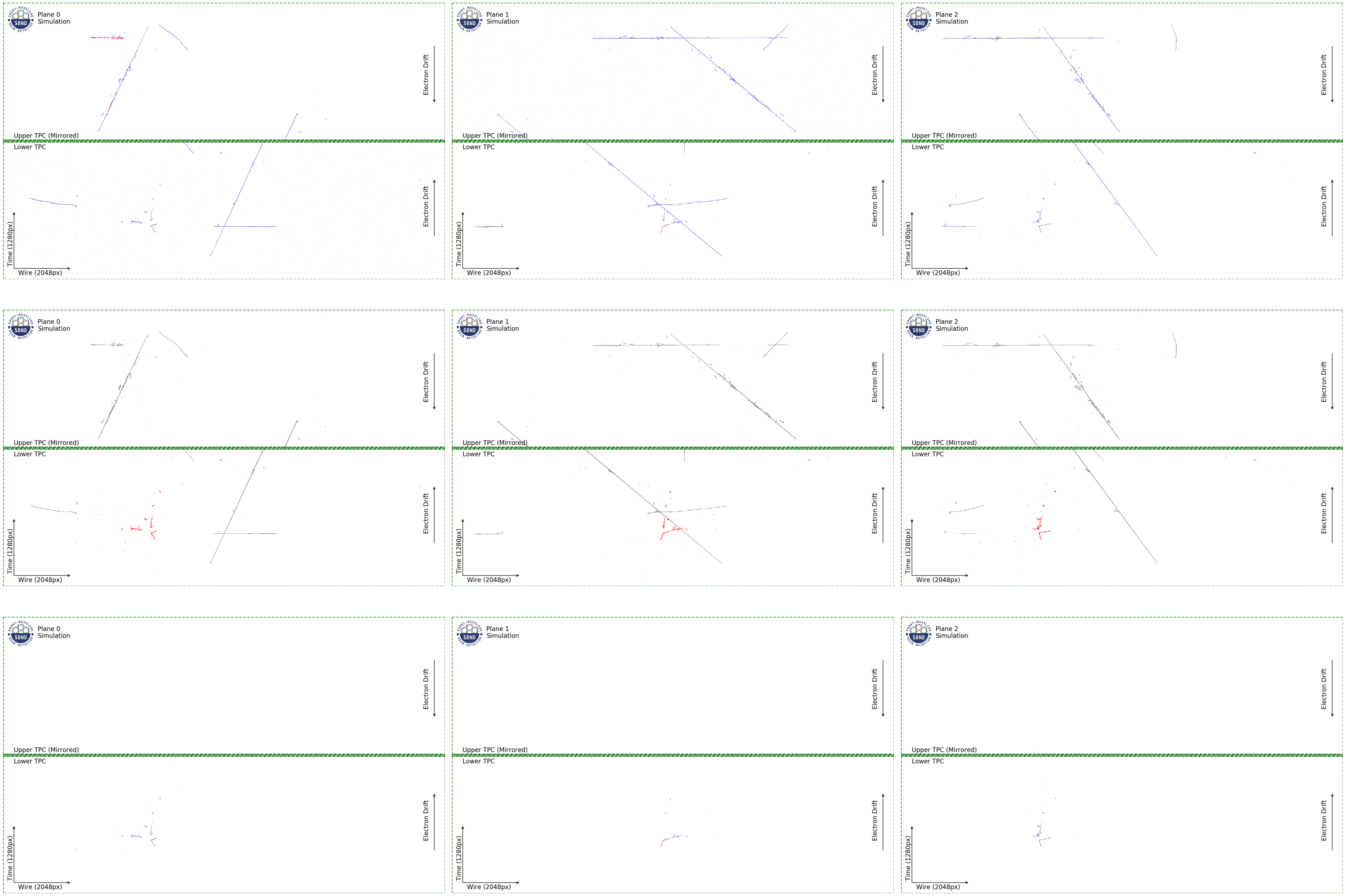

Figure S4: From left to right are the first, second and third projections. From top to bottom are the input images, the label images, and the selected neutrino pixels. This neutrino interaction has an overlapping cosmic muon in the middle column, degrading several pixels and causing the loss of a small track in the selection.
